# Meaningful Integration of Data from Heterogeneous Health Services and Home Environment Based on Ontology †

**DOI:** 10.3390/s19081747

**Published:** 2019-04-12

**Authors:** Cong Peng, Prashant Goswami

**Affiliations:** Department of Computer Science, Blekinge Institute of Technology, 371 79 Karlskrona, Sweden; prashant.goswami@bth.se

**Keywords:** health data integration, FHIR, WoT, REST, ontology, Semantic Web, Web service, eHealth, smart homes

## Abstract

The development of electronic health records, wearable devices, health applications and Internet of Things (IoT)-empowered smart homes is promoting various applications. It also makes health self-management much more feasible, which can partially mitigate one of the challenges that the current healthcare system is facing. Effective and convenient self-management of health requires the collaborative use of health data and home environment data from different services, devices, and even open data on the Web. Although health data interoperability standards including HL7 Fast Healthcare Interoperability Resources (FHIR) and IoT ontology including Semantic Sensor Network (SSN) have been developed and promoted, it is impossible for all the different categories of services to adopt the same standard in the near future. This study presents a method that applies Semantic Web technologies to integrate the health data and home environment data from heterogeneously built services and devices. We propose a Web Ontology Language (OWL)-based integration ontology that models health data from HL7 FHIR standard implemented services, normal Web services and Web of Things (WoT) services and Linked Data together with home environment data from formal ontology-described WoT services. It works on the resource integration layer of the layered integration architecture. An example use case with a prototype implementation shows that the proposed method successfully integrates the health data and home environment data into a resource graph. The integrated data are annotated with semantics and ontological links, which make them machine-understandable and cross-system reusable.

## 1. Introduction

The advent of the Internet of Things (IoT) is changing people’s lives and their integration with the surrounding environment. It is estimated that the number of connected IoT devices will outgrow the world population and increase to 50 billion by 2020 [1]. The technical evolution of IoT also stimulates the development of smart homes. It not only makes people’s daily living more convenient, but also can contribute solutions for challenges in healthcare system [2,3,4].

Chronic diseases have become one of the main threats to people’s health and a big challenge to healthcare systems all over the world [5]. The caring for chronic diseases requires long-term and periodical management by both the patients and healthcare staff. Therefore, it is better for the chronic disease patients to perform self-management at home due to the high cost and inconvenience of caring [6].

The development of information and communication technologies makes it much more feasible for the self-management of health. Electronic Health Record (EHR) systems have been adopted by many healthcare providers. Portable medical devices are used by patients for self-monitoring physiological parameters [6,7]. Many people today use wearable devices and health applications to record and manage their health [8,9,10]. Moreover, the IoT enabled smart home devices and health devices can provide a seamless environment for capturing data of a patient’s activities and vital signs [11].

The aforementioned systems, devices, and applications record a huge amount of health data from the patients. The collaborative use of the various health data has the potential to support chronic disease patients with more effective and convenient self-management [12,13]. Combining and collaborating the data of personal health management with home environment and indoor exercise can provide a comprehensive overview and an increased understanding, which encourage the caring of one’s health management and lead to focused behavior changes [14].

Unfortunately, all these health data became data silos, which can only be used in their own places with very limited outside collaboration. One reason behind this situation is that the systems and devices holding these health data are heterogeneously built. Also, the protocols, e.g., Bluetooth, ZigBee, and RFID, used to communicate with the IoT devices are varied, and many of the devices and service applications rely on certain platforms or technology, which restricts the integration of these devices [15,16].

Web services technologies have promoted the interoperability of various software applications running on distributed and diversified systems. The concept of the Web of Things (WoT) was then proposed to access, interconnect and operate IoT devices on upper-layer protocols by applying standard Web technologies, and abstract services or resources on the Web [17,18,19]. However, the lack of common standards adoption still makes it problematic for integrating health data with heterogeneous data models, especially among services from different domains of eHealth and IoT.

Various work has been done on the health informatics standards. Among these, the Fast Healthcare Interoperability Resources (FHIR) created by the Health Level Seven International (HL7) organization is regarded as the next generation of health information interoperability framework that combines the previous standards’ features [20,21]. It leverages common Web standards, which includes applying REpresentational State Transfer (REST) architectural style and JSON serialization format, besides the previous supported XML, as interfaces for health information exchange [22].

The FHIR working group is also working on the FHIR Linked Data module that uses Semantic Web technologies such as Resource Description Framework (RDF) and Web Ontology Language (OWL) to enhance its semantic expression capability and to facilitate inference and data linkage across datasets [20,22]. Semantic Web of Things (SWoT) was also proposed to enable a wide-scale interoperability, integration, composition and even more by annotating semantics to the representation of the things on the Web [23,24]. Various vocabularies and ontologies have been proposed. The widely used contributions include, but are not limited to, the IoT extension of Schema.org [25], Semantic Sensor Network (SSN) and Sensor, Observation, Sample, and Actuator (SOSA) Ontology [26,27], and Web of Things (WoT) Thing Description [28].

The work in this paper proposes a method, which follows the path of FHIR to apply Semantic Web technologies and the REST resource model, to integrate health data and home environment data from heterogeneously built services as linked resources. The integration method aims to semantically link the health services and WoT devices that adopted HL7 FHIR, described by formal ontologies, implemented RESTful Web APIs or published as Linked Data, i.e., services with different levels of interoperability. It is to make the integration method compatible with the real-world service environment. The heterogeneous data are modeled as conceptual information resources by using the Linked Health Resources (LHR) ontology. It makes the entire method as a consolidated framework that aggregates health and home environment data from different sources and integrates them as a data resource graph, in the way of RDF, for upper-level collaborative use. The integrated data are machine-understandable and cross-system reusable thanks to the semantics and ontology links contained in RDF.

It needs to be clarified that this paper builds on our previous work [29] and includes an extended method with more detailed description. To achieve an enhanced support and a more well-covered self-management of health, the method of health data integration is extended to include the home environment data, besides the personal health data in the previous work. The modeling of health resource and the layered integration architecture are extended to cover data resources from WoT services. The previously proposed integration ontology is also extended to integrate data described by IoT and WoT ontologies. Besides health resource classes, home environment resource classes are added to link to ontologies include SSN, SOSA, and WoT Thing Description.

The structure of the rest of the paper is as follows. Section 2 introduces the previous works on integrating health data. In Section 3, we present the method, which includes the health resource modeling, LHR ontology, and the integration architecture. Section 4 presents an example use case to demonstrate the health data integration process by the proposed method. Finally, we conclude our work of this paper in Section 5.

## 2. Related Work

Interoperability is important and closely related to health data integration. Various interoperability standards (HL7 standards, Digital Imaging and Communications in Medicine (DICOM), Clinical Information Modeling Initiative (CIMI), etc.) and clinical coding systems (SNOMED-CT, ICD, Logical Observation Identifiers Names and Codes (LOINC), etc.) have been introduced to improve the interoperability in eHealth [30].

Much work has been done on integrating health data to support healthcare monitoring and decision making for either healthcare professionals or patients. To enable healthcare providers to remotely monitor and interpret health trends of diabetes patients, a method to integrate blood glucose data recorded from a patient-facing device to an EHR system was presented in [6]. The integration was achieved by transmitting the glucose data to the device vendor’s iOS mobile phone application. It shares the data with the Apple HealthKit, which then sends the data to the EHR system. This solution is locked to the iOS platform since it depends on the Apple HealthKit.

V. Gay and P. Leijdekkers demonstrated a mobile application approach to aggregate health and fitness data to enable interoperability [9]. It was achieved by an Android application they developed with third-party partners to connect with wearable devices, EHR systems, and other applications. A patient-centric mobile healthcare system that integrates data from body sensors was presented in [31]. The integration was implemented by leveraging a RESTful Web service on the application layer of the system to enable data sharing among applications. Seo et al. proposed an information fusion method, which is platform independent that accesses data by using only REST Web APIs [32]. The sensor and activity models of the information fusion method classify and aggregate data from different types of sensors including physical, activity, and social sensors.

There are also works done with Semantic Web technologies to enable health data integration with semantics. SENHANCE is a framework proposed by I. Pagkalos and L. Petrou to integrate patient self-reported health data on social networks with hardware sensor observation data supported by Semantic Web technologies [7]. It uses Web APIs provided by social network platforms such as Facebook, Nike+ Running and MyFitnessPal to retrieve data include activities, fitness, and food journal. The social networks data are then modeled as human sensor observations together with hardware sensor observations described by their proposed ontology and stored in RDF. The proposed ontology is constructed in OWL and interlinked with external ontologies include Sensor Network (SSN) ontology [26], Friend Of A Friend (FOAF) [33] and PROV-O Ontology [34]. B. Tilahun et al. presented a Linked Data-based system to retrieve and visualize heterogeneous health data in a flexible and reusable way [35]. The system used a set of Semantic Web technologies include RDF, Fuseki and SPARQL (SPARQL Protocol and RDF Query Language) for data representation, storage, and query.

To integrate functionalities of different devices for supporting home-based care, an integration platform architecture was presented by Y. Trinugroho, F. Reichert and R. Fensli [36]. A smart home ontology that covered the modeling of person, device, and context was proposed to enhance the reasoning process. To give semantics for the exchanged data between devices and systems in the home context, another smart home ontology was proposed in [3]. In addition, for the purpose of increasing the usability, the integration of smart home data with other data sources was also explored by applying Linked Data principles.

R. Reda et al. proposed an ontology-based semantic data annotation framework to describe sensor data and to facilitate interoperability and health data integration [37]. The framework consists of two components, which are IoT Fitness Ontology (IFO) and mapping process. The IFO ontology aims to semantically describe the most common concepts and their relationships within the IoT fitness and wellness devices and corresponding health application services. It also links to external ontologies such as SNOMED-CT for wide semantic integration. The mapping process applies RDF Mapping Language (RML) [38] to generate RDF graph from common formats including CSV, XML, and JSON. In addition, the market-available systems Fitbit, Apple Health, and Nokia Health are referred for defining the mapping specifications. A HealthIoT ontology was proposed by A. Rhayem et al. to describe the semantics of connected objects and data in Internet of Medical Things, which can facilitate semantic rule reasoning to assist clinical decision making [39].

Despite several works exploring the use of Semantic Web technologies to integrate data from health service and WoT home environment devices, the integration among the ordinary health services, the services implemented with the FHIR standard, and the WoT services described by formal ontologies remains unexplored. In addition, the existing works lack flexibility in the framework or ontology model. It makes them intolerant to data with different interoperability levels, which is the actual situation in the real-world service environment. Therefore, our proposed method tries to semantically integrate data from the heterogeneous services, and to keep the flexibility for the tolerance of data with different interoperability levels.

## 3. Method

The LHR framework intends to integrate 4 categories of health-related data sources: (1) FHIR-enabled health services, (2) health services and WoT services implemented Web APIs (satisfy certain REST constraints), (3) formal ontology described WoT services and (4) health-related Linked Data. The WoT services provide data or interactions to the health-related devices or smart home environment. The data of smart home environment are considered as health-related resource as well, since they are related to a person’s health management. The 4 categories cover most available health-related services and data, either for the current stage or the near future development trends.

The following subsections start with explaining the natural similarities of data resources from various services, and then introduce our view on the modeling of health and home environment data resources. An integration ontology and a layered integration architecture are then presented.

### 3.1. The Nature of Health Resource

Today most of the health services that provide data access are delivered in the form of Web APIs, which usually serve data in JSON or XML serialization format via HTTP methods. Most of these Web APIs claim to be RESTful services. Though in many cases they only follow a few of the REST constraints, which makes them actually not RESTful services. However, most of the services follow one of the fundamental elements of REST to organize the accessible data as resources, which is the unit of information in REST architectural style [40]. It makes them capable of being modeled together under the LHR framework.

A WoT object implemented Web API means that it uses HTTP as interface and URL as resource locator or identifier [19,41]. For a WoT object that is connected to a Web service and the data or interaction of that WoT object are accessed via the connected Web service, it can be regarded as the same as an ordinary Web resource. For a local WoT object, i.e., a WoT object that itself serves as a local Web server or is connected to a local gateway instead of connected to a Web service, it then can be regarded as a device. However, the retrieved data representation of the WoT object is eventually a data resource.

To improve the implementability and to be more developer-friendly, the HL7 FHIR standard leverages the common Web technologies and concepts. FHIR therefore follows the REST architectural style as well, and is built upon a set of resources. Resource in the case of FHIR means “a collection of information models that define the data elements, constraints and relationships for the objects relevant to healthcare” [20]. The objects that are modeled as resources include *Patient*, *Observation*, *OperationOutcome* and so on. Each resource is defined in a certain structure with references to other resources, and represented in XML, JSON, and an additional RDF serializable format Turtle.

Linked Data is a practice of publishing structured and interlinked data with semantic meanings on the Web to make it a Web of data [42]. It was proposed by Tim Berners-Lee as an application of Semantic Web technologies. The Linked Data rules align well with some of the REST constraints. There have been many works on linking REST Web services and Linked Data [43,44]. Different levels of practices exist on publishing Linked Data according to the 5-star rating system developed by Berners-Lee [42]. A proper practice of publishing Linked Data should identify interlinked things (data items or real-world entities represented on the Web) with HTTP Uniform Resource Identifiers (URIs) and serve corresponding information in RDF serializable formats or SPARQL query service.

Accordingly, we can regard anything identified by an HTTP URI as a resource, i.e., a node in an RDF graph. In addition, the resource identified by the root path of an HTTP URI can be regarded as a service, i.e., a root node of an RDF graph.

### 3.2. Modeling of Health Resource

**Figure 1:**
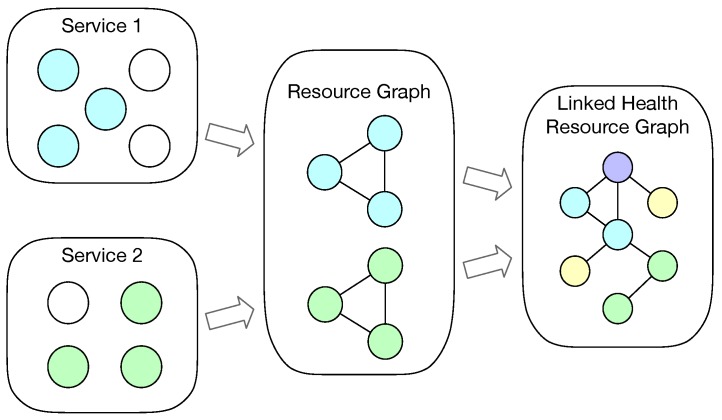
A simple overview of the integration process (each circle stands for a resource). Resources are aggregated from services into semantic resource graphs, which are then integrated as a Linked Health Resource graph.

Figure 1 shows a simple overview of the process that resources from different services being integrated into a health resource graph. Based on the nature of health Web services, WoT service, and Linked Data services, each service contains a set of resources, irrespective if the service is implemented with formal description model (e.g., FHIR, SSN ontology) or not. Let $S_{i}$ be a health or WoT service, where $S_{i}=\sum R_{j}$ denotes that service $S_{i}$ serves a set of resources $R_{j}$.

We assume that a person $P_{k}$ has several health or WoT services, which have been integrated together into his or her health resource graph $G(P_{k})$. A health resource graph means a set of health or home environment information being modeled as resources with relationships, which maps to the RDF graph data model. Then $G\left(P_{k}\right)=\sum S_{i}=\sum \sum R_{ij}$ denotes that a person’s health and home environment status can be described by sets of health and home environment resources from a collection of services. The sets of health and home environment resources can be represented in an integrated resource graph.

Furthermore, a resource itself contains several objects:

- A resource may have interlinked (subordinate or related) resources *R_link_*.
- A resource may have data items *D_r_* that describe certain status about a person, for example profile data, observation data, etc.
- A resource can be annotated by a set of *n* semantic tags *T_r_*, where *n* ∈ [0,∞].

A resource can therefore be represented as a tuple $R_{j}=\left(D_{r},R_{link},T_{r}\right)$.

### 3.3. Linked Health Resource Ontology

**Figure 2:**
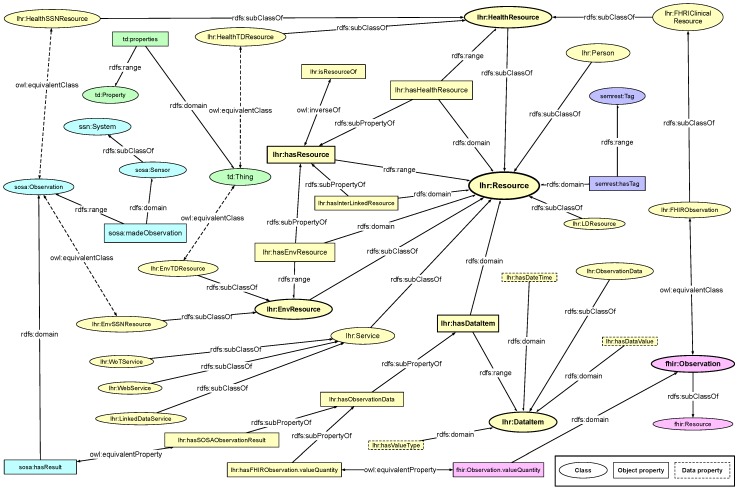
Linked Health Resource Ontology (with vital objects). The integrated health services, home environment devices and resources are modeled as sub-classes of the *lhr:Resource* class, which is the fundamental class in this ontology. Certain equivalent classes and properties are mapped to FHIR, SSN, SOSA, and WoT Thing Description for integration.

When investigating the very nature of the relationships among person, service, and resource, we can see that a service as a unit also has resources that are interlinked, which is the same as a resource. It makes a service to be a resource. By the same logic, a person can be regarded as a resource as well. Therefore, we model the three types of objects all as sub-classes of class *lhr:Resource* under the LHR ontology, where the basic relationships among them being *lhr:hasResource* and *lhr:isResourceOf*. Figure 2 shows the main classes and properties in the LHR ontology. It is built via the W3C standards, the RDF Schema (RDFS) data modeling vocabulary [45] and the OWL ontology language [46].

As stated before, the class *lhr:Resource* is the super-class of all the involved resources. The class *lhr:Service* is a sub-class of *lhr:Resource*, and represents a set of resources in a relatively separated territory to indicate a source of health data. The class *lhr:Person* represents a person who owns all the resources. The class *lhr:HealthResource* represents all a person instance’s health resources that are interlinked from ordinary health Web services and FHIR implemented services. The class *lhr:EnvResource* represents all a person instance’s home environment resources that are interlinked from ordinary WoT services and formal description implemented services. Usually Linked Data are not about only a single person. Therefore, resources from Linked Data are classified as the more generic class *lhr:LDResource*.

To link the FHIR health-related resources, which are categorized as clinical resources in FHIR, we map those clinical resources into the LHR ontology. For instance, the *fhir:Observation* is mapped to *lhr:FHIRObservation*, which is a sub-class of *lhr:HealthResource*. Health resources from other ordinary Web services or Linked Data sets can also map from a class that have been defined in other ontologies or vocabularies to a sub-class of *lhr:HealthResource*. Alternatively, the origin resource can instantiate directly from *lhr:HealthResource* if there is no embedded class.

To link the WoT resources, we also map the objects in external IoT and WoT vocabulary and ontology into the LHR ontology. The *ssn:System* and *sosa:Sensor* in SSN and SOSA ontology [27] are mapped to *lhr:Service* and *lhr:Resource*. The *sosa:Observation* can be made as an equivalent class to either *lhr:HealthSSNResource* or *lhr:EnvSSNResource*, which are sub-classes to *lhr:HealthResource* and *lhr:EnvResource*, respectively. A resource from a WoT device could be either health-related or home environment related depending on the actual type of data.

Object properties around the class *lhr:Resource* and its sub-classes are the transitive property *lhr:hasResource* and its sub-properties include *lhr:hasHealthResource*, *lhr:hasLDResource* and *lhr:hasInterLinkedResource*. Among which, the property *lhr:hasHealthResource* has *lhr:Resource* as its domain and *lhr:HealthResource* as its range, the property *lhr:hasLDResource* has *lhr:Resource* as its domain and *lhr:LDResource* as its range. The object property *sosa:madeObservation* in SSN and SOSA ontology is mapped to a sub-class of *lhr:hasResource*.

The class *lhr:DataItem* is used to represent the data model of a *lhr:Resource* if it has no predefined model, and class *lhr:ObservationData* is used to represent the health data model of a *lhr:HealthResource*. The resources from FHIR service have predefined data models in RDF, therefore, the object of which are mapped to an instance of *lhr:ObservationData*. The relation is represented by a sub-property of the object property *lhr:hasObservationData*. The same logic applies to the resources from SSN and SOSA implemented service. The *sosa:hasResult* object property is made as an equivalent property to *lhr:hasSOSAObservationResult*, which is also a sub-property of *lhr:hasObservationData*.

The LHR ontology is intended to be made flexible for tolerance of heterogeneity by adding extension. Data resource described by other ontology could also be linked to LHR ontology in the same way as FHIR and SSN by adding extension of mapping between classes. The mapping extension is described in the same way as LHR ontology by using RDFS and OWL. For instance, the *td:Thing* in WoT Thing Description [28] and the *semrest:Representation* in SemREST vocabulary [47] could be mapped to either *lhr:HealthResource* or *lhr:EnvResource* as well. The *semrest:dataItem* and *semrest:hasValue* could be mapped to *lhr:hasDataItem* and *lhr:hasDataValue*, respectively.

With the sub-class relations and transitive sub-property relations defined in the LHR ontology, we can easily infer that an instance of *lhr:Person* has a set of instances of *lhr:HealthResource* and *lhr:EnvResource* being linked together without complex inference rules.

### 3.4. Layered Architecture towards Integration

**Figure 3:**
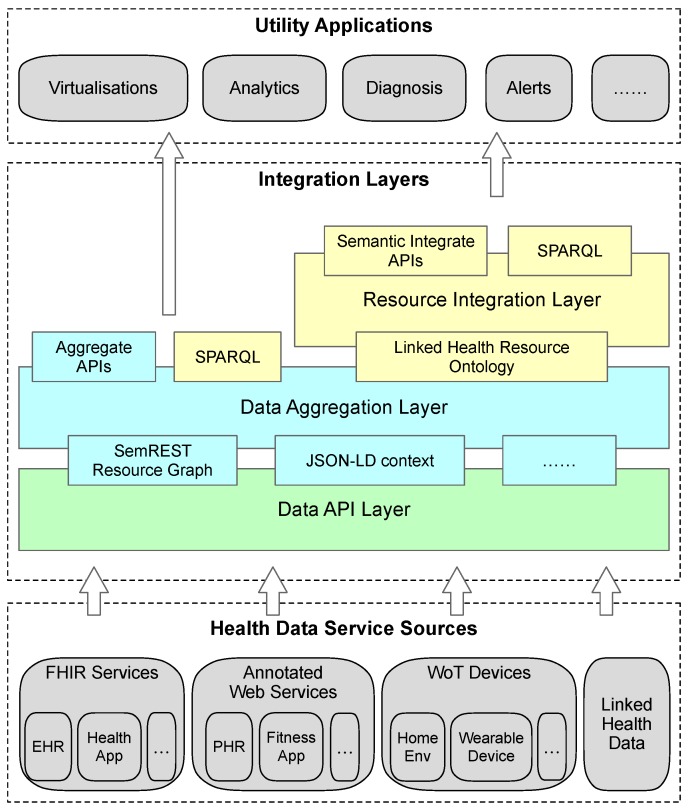
Layered architecture of data integration with the Linked Health Resource. The ontology modeling process works on the resource integration layer, which receives the aggregated resource graph from the data aggregation layer.

For integrating different health data into one ontological model with LHR, we need to firstly aggregate the health data together from different services. Figure 3 illustrates the layered architecture for integrating health data from different categories of services, as aforementioned that the LHR framework intends to integrate, into an LHR ontological model.

From bottom to top, the first layer is the data API layer. This layer simply requests data from services via APIs, e.g., Web APIs or Linked Data APIs. The retrieved data resources are then aggregated together into a semantic resource graph on the data aggregation layer. To be effectively aggregated into the semantic resource graph, it is necessary for the ordinary Web and WoT services to have some simple semantic annotations with commonly used vocabularies. Besides the Semantic RESource Tagging (SemREST) in [47,48], JSON-LD context embedding to ordinary JSON representation [49] or other Semantic Web service annotation methods such as the ones presented in [38,50,51,52] could also be used to semantically annotate the resource. For the concerned parts of the data resource to be integrated on the integration layer, the semantic annotations that added to the data representation could directly link to the classes and properties in LHR ontology. Alternatively, the aforementioned mapping extension could be added to the LHR ontology.

The following three categories of services can be aggregated into a semantic resource graph without much effort. The FHIR implemented services follow the FHIR standards, therefore, the data resources come with standard structure and semantics. Some of the services even implemented FHIR/RDF representations. When the data resources from a WoT service that follows a formal description ontology, e.g., SSN ontology [27], and the data resource of which is represented in an RDF serializable representation, it can be aggregated directly into a semantic resource graph as well. Data retrieved from Linked Data services are already RDF serializable if they were implemented properly.

The semantic resource graph aggregated with the retrieved data is then sent to the information resource integration layer. The LHR ontology is used to extract health resources from the resource graph for integration. One thing that needs to be noted is that the concept *resource* in the semantic resource graph from the data aggregation layer is slightly different from the *lhr:Resource* in LHR ontology. The former *resource* maps to a resource in a REST Web service, it contains functional meta information of its Web API. Only its representation unit (it is determined by the semantic annotation embedded in the representation or the mapping extension) will be passed on to the integration layer as an instance of *lhr:HealthResource* or *lhr:EnvResource*.

On top of the information resource integration layer there could be semantic APIs that use the health resource instances of *lhr:Resource* and the contained *lhr:DataItem*. Providing APIs on modelized resources in this manner has the potential to provide a unified interface to use the health data from different sources in the wrapped model for applications like health data visualization, analytics, and so on. Alternatively, applications could also use health data in the form of semantic resource graph above the data aggregation layer. Since all the resources are represented in RDF on both the aggregation layer and integration layer, it can serve SPARQL queries directly.

**Figure 4:**
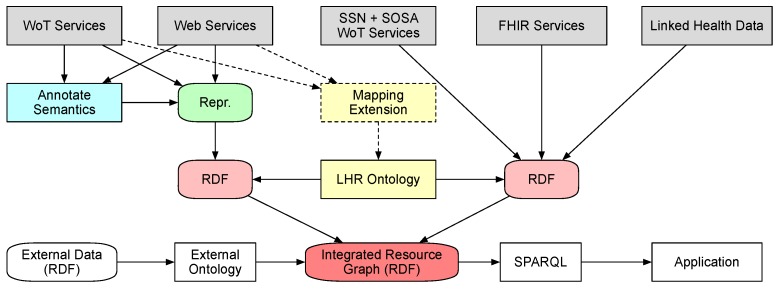
A data-centric view of the integration process.

To have a clearer view on how the data flow from hosting services to resource graph, Figure 4 presents a data-centric view of the integration process. The data are hosted at the services initially. The data from ordinary Web services and WoT services need to be annotated with semantics with commonly used vocabularies. The hosting service could optionally provide a mapping extension to indicate the mappings between the types of data units in the representation to the classes and properties in LHR ontology. In addition, then they could be published as different representation such as JSON and XML, as long as the concerned parts of them can be transformed into RDF with the annotated semantics. The data from services that represent data with formal vocabulary and ontology are published as RDF serialization formats such as RDF/Turtle, RDF/XML and JSON-LD. After retrieval by the integration service, the data are passed from the hosting services to the integration service as separate RDF documents. By using the LHR ontology and the optional mapping extension, the data are then integrated into a single resource graph represented in RDF. External data could be referenced through ontology links with the resource graph. The integrated data queried by applications through SPARQL queries.

## 4. Use Case of Integration

To demonstrate the LHR health data integration process, an example use case is presented in this section. It is depicted as an idealized scenario where it leaves aside some details like the authentication and invocation of services. The demonstration prototype is implemented as two parts: the *aggregation layer* is implemented in Python with the RDFlib package [53], and the *integration layer* is implemented in Java with the Jena framework [54]. The example use case focuses on the integration layer since it is the main content of this paper. Therefore, the working process of aggregation is omitted here.

The use case scenario is that a person named Alice is actively managing her own health condition. She has taken some medical examination at a healthcare center, which provides health data access via an HL7 FHIR implemented service. At the same time, she has been using a Fitbit health band and its application service to track her daily activities and life style data for some time. She is also using a home environment WoT device to measure indoor temperature and humidity before she exercises at home if the steps she walked is not enough. To have a periodical comprehensive view, she wants to integrate her *blood glucose* examination data from the healthcare center, her data of *daily steps performed*, *exercises performed* from Fitbit Web service, and her home environment *temperature* and *humidity* measures from the WoT device together.

For demonstration purpose, the data sample of *blood glucose* uses the FHIR official observation example [55]. Listing 1 shows the simplified resource of Alice’s *blood glucose* observation data in RDF/Turtle format. This resource is passed through the data aggregation layer to the integration layer directly since the FHIR observation resource is an instance of the class *fhir:Observation* that maps to *lhr:FHIRObservation*.



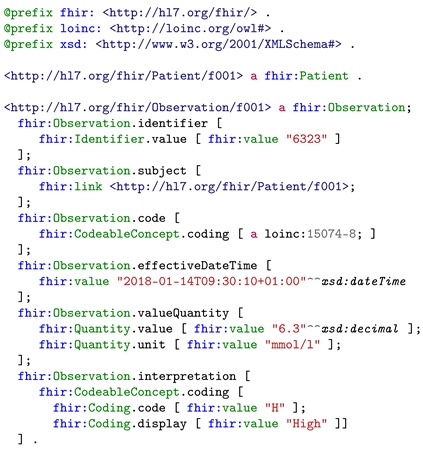

**Listing 1.** Simplified FHIR observation of blood glucose example represented in RDF/Turtle format. The observation resource contains the value, the effective date time, and the LOINC code indicating the medical terminology.

Next we are going to integrate the *daily steps performed* data. Fitbit provides Web APIs to access the data recorded by their wearable devices. For integrating the data resource from Fitbit Web service into the LHR model, the semantic annotation method SemREST [47] is applied to annotate its service description and aggregate the resource representation on the data aggregation layer. SemREST is also a method based on JSON-LD context embedding but with principles for annotations. The generated semantic resource graph of Fitbit *daily steps performed* is partly shown in Listing 2 in RDF/Turtle format. It is then passed on to the integration layer together with the mapping extension shows in Listing 3. The mapping extension describes the mapping relationship between SemREST and LHR.

Alice’s home environment WoT device applies SSN and SOSA ontology to represent the *temperature* and *humidity* data. Listing 4 shows the simplified resource of observation data in RDF/Turtle format. This resource is passed through the data aggregation layer to the integration layer directly since the SOSA sensor observation resource is an instance of the class *sosa:Observation* that maps to *lhr:EnvSSNResource*, which is a sub-class of *lhr:EnvResource*.

On the integration layer, the coming resources are integrated using the LHR ontology. As mentioned before, the FHIR *blood glucose* observation resource is an instance of *lhr:FHIRObservation*, which is an equivalent class to *fhir:Observation*. For the ease of accessing the data value of an FHIR observation resource with LHR, the property *fhir:Observation.valueQuantity* is mapped to the corresponding object property *lhr:hasFHIRObservation.valueQuantity*, which is a sub-class of *lhr:hasObservationData*. In addition, the *fhir:Observation.effectiveDateTime* is mapped to the corresponding data property *lhr:hasObservationDateTime*. Other objects could be accessed directly through the *fhir:Observation* instance, including the *fhir:Observation.code*, *fhir:Observation.interpretation* and *fhir:Observation.referenceRange*. Furthermore, for a more convenient use for upper-level APIs, the object of *fhir:Observation.code* could be mapped to *lhr:DataItem*’s value type to indicate the semantic meaning of the data value.



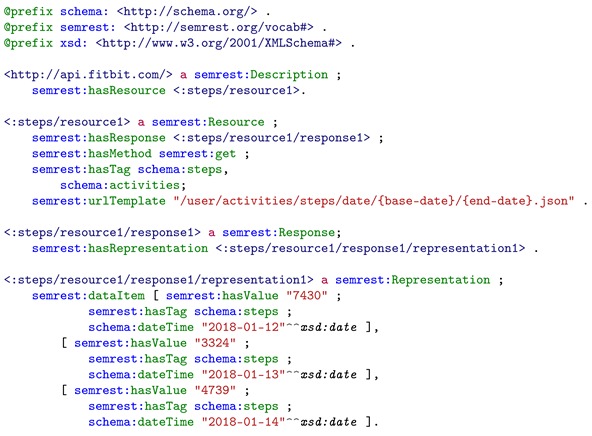

**Listing 2.** Simplified resource graph of Fitbit steps resource generated by SemREST and represented in RDF/Turtle format. The resource contains each day’s step count, date, time, and the steps term in schema.org as a tag indicating the semantic meaning.

For the Fitbit *daily steps performed* resource, the *semrest:Representation* instance *fitbit:representation1* in the resource graph from the aggregation layer is instantiated as a *lhr:HealthResource*, indicated by the mapping extension. Since the *steps* data has been annotated as the property *semrest:dataItem*’s objects, so it is mapped to the objects of *lhr:hasObservationData*, a sub-property of *lhr:hasDataItem*, to this LHR health resource. The data value, date, time, and tag are also mapped to the objects in the ontology for the upper-level APIs.

The home environment *temperature* and *humidity* observation resources are made as instances of *lhr:EnvSSNResource*, which is an equivalent class to *sosa:Observation*. For the ease of accessing the data value of an SOSA observation resource with LHR, the property *sosa:hasResult* is mapped to the corresponding object property *lhr:hasSOSAObservationResult*, which is a sub-class of *lhr:hasObservationData*. In addition, the *sosa:resultTime* is mapped to the corresponding data property *lhr:hasObservationDateTime*. Other objects could be accessed directly through the *sosa:Observation* instance, including *sosa:madeBySensor* and *sosa:observedProperty*.

**Figure 5:**
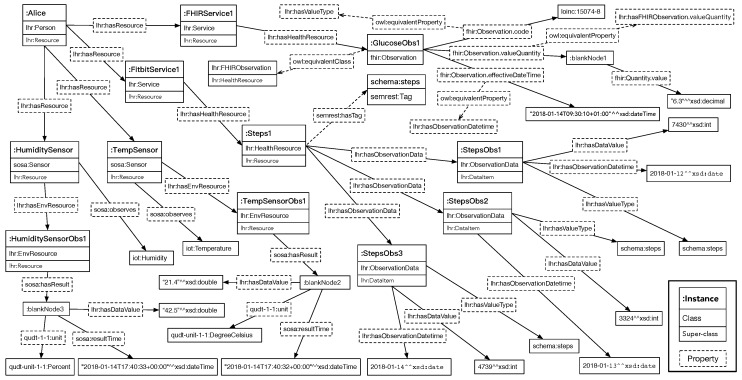
Graphical presentation of Alice’s integrated health and home environment resources under the LHR ontology, where each instance’s class or property is presented with super-class or super-property.

The RDF of the integrated health and environment resources is depicted graphically in Figure 5 with relatively important objects. Each square block represents an instance with its class and super-class. Instances are linked by the object properties and data properties. Since there exist many equivalent classes and properties, the ones considered more appropriate are placed in the graph to avoid information redundancy.

**Figure 6:**
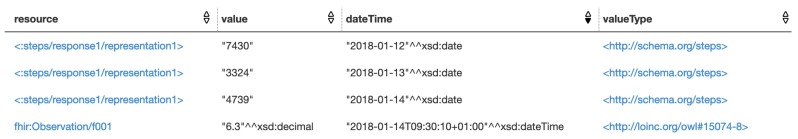
The result of the example SPARQL query for health resources, all the Alice’s health resources that were modeled as *lhr:HealtResource* are retrieved with their data value, data time, and value type.

As one of the methods that can use the integrated health resources, SPARQL queries is executed to demonstrate that the different data resources are integrated and can be used together. Apache Jena Fuseki is used to serve the integrated resource graph as SPARQL server. The queries are executed by using its Web-based client. The SPARQL query in Listing 5 is executed to query for Alice’s health resources. The purpose of this SPARQL query is to test that if the *blood glucose* FHIR data resource and the *daily steps performed* Fitbit data resource are successfully integrated and can be retrieved in a single SPARQL query. The query result is presented in Figure 6.



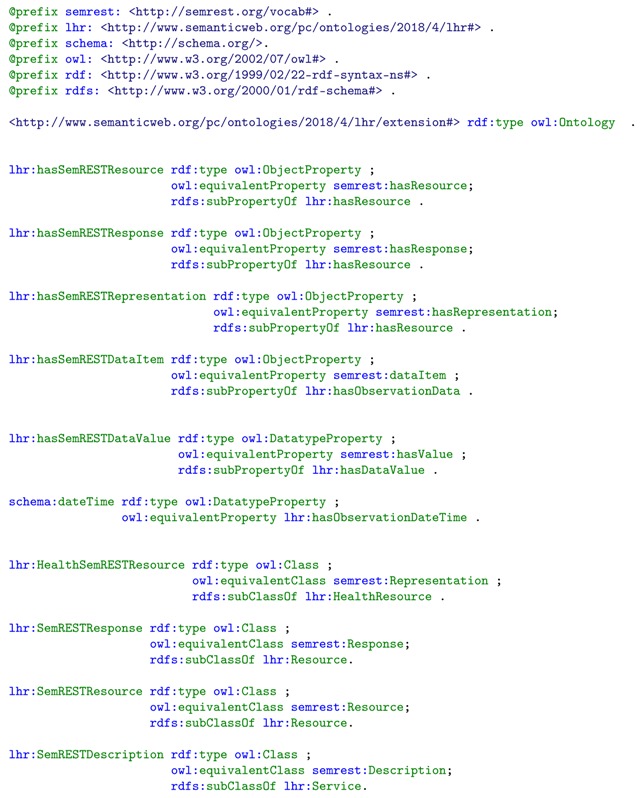

**Listing 3.** LHR mapping extension for classes and properties in SemREST.



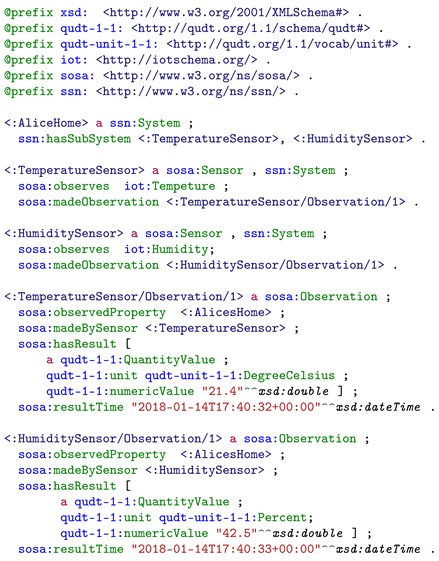

**Listing 4.** Simplified resource representation of home environment temperature and humidity represented with SSN and SOSA ontology in RDF/Turtle format.



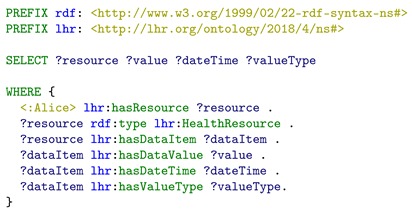

**Listing 5.** Example SPARQL query to retrieve integrated LHR health resources with their data value, date, time, and value type.

We can see that both the FHIR observation resource and the Fitbit resource are retrieved, since both the two types of resources were modeled as *lhr:HealthResource* directly (the Fitbit *daily steps performed* data resource <:steps/response1/representation1>), or through sub-class relation (the FHIR *blood glucose* data resource <fhir:Observation/f001>, through the mapping between class *lhr:FHIRObservation* and *fhir:Observation*). Within the integrated resource graph, the value, date time, and value type have been mapped into the LHR ontology as well (through the class *lhr:DataItem*), so we can query them through the properties of *lhr:DataItem*. The value type of each data item is actually an URI. It not only works as the value type’s dereferenceable URI for indicating the semantic meaning, but also integrates the resource under LHR with other Linked Data, where the same URI were referenced. The query result shows that the FHIR *blood glucose* data resource and the Fitbit *daily steps performed* data resource have been successfully integrated and are retrieved by a single SPARQL query.

**Figure 7:**
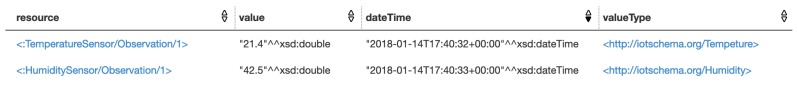
The result of the example SPARQL query, all the Alice’s home environment resources that were modeled as *lhr:EnvResource* are retrieved with their data value, data time, and value type.

From the data queried, Alice finds that the steps walked today is not enough, so she decides to do some exercise at home. To have a better performance and outcome for the exercise, Alice wants to make sure that the home environment temperature and humidity are within a comfortable range. So, another SPARQL query in Listing 6 is executed to query for the home environment resources. The purpose of this SPARQL query is to test that if the WoT *temperature* and *humidity* data resource are successfully integrated into the resource graph and can be retrieved in a single SPARQL query. The query result is presented in Figure 7. We can see that both the humidity and temperature resources are retrieved, since both were modeled as *lhr:EnvResource*, through the mapping between *lhr:EnvSSNResource* and *sosa:Observation*. In addition, the value, date, time, and value type also have been mapped into the LHR ontology (through the class *lhr:DataItem*), so they can be retrieved in this query. The query result shows that the WoT *temperature* and *humidity* data resource have been successfully integrated into the resource graph and are retrieved by a single SPARQL query.

To present the experiment results in a simple clear way and to avoid repetition, the health resources, and home environment resources are retrieved in two separate SPARQL queries. They can be certainly retrieved together in a single query if needed, since all the data resources are contained in one integrated resource graph as RDF.

To demonstrate the health data integration in a simple view, the example use case was performed far from a realistic way of health self-management. However, this integration method can work in real cases since the data integration working process is the same.



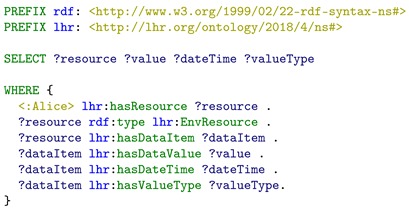

**Listing 6.** Example SPARQL query to retrieve integrated LHR home environment resources with their data value, date, time, and value type.

## 5. Conclusions

In this paper, we presented a method to integrate HL7 FHIR interoperability standard implemented health services, SSN and SOSA ontology-described WoT services, ordinary Web-based health services, and WoT services with a few semantic annotations. The proposed OWL-based LHR ontology modeled different categories of health resources and environment resources together in a clear and simple way for upper-level unified use. Thanks to the application of Semantic Web technologies and the well-aligned conceptual model with Linked Data, this method can also integrate health resources from the Linked Open Data on the Web without much effort.

With the capability to integrate different categories of health services and WoT services, which usually contain data from healthcare providers, consumer-facing products, and health research, the presented method has the potential to support self-management of health especially for people with chronic diseases. In addition, the data integrated through this method are annotated with semantics and ontological links, which make them machine-understandable and reusable across systems. It provides possibilities for imaginative applications with the collaborative use in the semantic-rich and cross-sources health and home environment data. To the best of our knowledge, the work in this paper is the first attempt to semantically integrate data from the aforementioned heterogeneous services. In addition, it also tries to keep the flexibility for tolerance of data with different interoperability levels, which is the actual situation of the real-world service environment.

To achieve a satisfiable interoperable integration, it would be ideal that the involved health services implement interoperability standards such as HL7 FHIR, and WoT devices could implement description ontology such as SSN and SOSA. However, obstacles including services with legacy system problems exist. That makes it impossible for all the services to realize in the near future. Diversity may exist among the different health services. The approaches such as the one we proposed can act as a bridge to connect the heterogeneously implemented services. Integrating health data as an ontological model with semantic meaning is beneficial for upper-level use in a more unified way.

Limitations, however, exist in this work. Though this method tries to make the integration easy and flexible, it still requires extra semantic annotation by the service providers for integrating the ordinary Web and WoT services. Ontology mapping by extra links to LHR ontology or mapping extension is also needed for the resources from the data aggregation layer to be integrated on the integration layer. Otherwise, it is unable to identify which part of the resource should be integrated.

For the current integration method using LHR ontology, it is possible to lose some of the context or related information by only looking at the data in integrated resource graph. It is difficult for LHR ontology to map to the many different data models and context information such as the unit of a data value. It is important to keep the access to the data resource that has all the information for possible look up. If the service annotates all the relevant data field with semantics, then it would be probable to have the complete data on the aggregation layer available for query with semantics. The more semantic annotations embedded in a resource’s representation, the more machine-understandable integration it will be, which can promote more useful applications. For both building new ontology and annotating semantics to data, reusing existing ontologies is recommended and more pragmatic on account of reducing unnecessary repetitive work and increasing linkage of data on the Web. However, difficulties may occur due to incomplete-matched concepts or insufficient explanation for certain terms.

In future work, we plan to evaluate this method with more real-world services for improvement and validation. In addition, the upper-level semantic-use APIs and applications can be explored for the collaborative use of health data.

## References

[1] Evans D. (2011). The Internet of Things: How the Next Evolution of the Internet is Changing Everything.

[2] Gietzelt M., Von Bargen T., Kohlmann M., Marschollek M., Schwartze J., Song B., Wagner M., Wolf K.H., Haux R. (2014). Home-centered health-enabling technologies and regional health information systems an integration approach based on international standards. Methods Inf. Med..

[3] Puustjärvi J., Puustjärvi L. (2015). The Role of Smart Data in Smart Home: Health Monitoring Case. Procedia Comput. Sci..

[4] Wu W., Pirbhulal S., Sangaiah A.K., Mukhopadhyay S.C., Li G. (2018). Optimization of signal quality over comfortability of textile electrodes for ECG monitoring in fog computing based medical applications. Future Gener. Comput. Syst..

[5] Daar A.S., Singer P.A., Leah Persad D., Pramming S.K., Matthews D.R., Beaglehole R., Bernstein A., Borysiewicz L.K., Colagiuri S., Ganguly N. (2007). Grand challenges in chronic non-communicable diseases. Nature.

[6] Kumar R.B., Goren N.D., Stark D.E., Wall D.P., Longhurst C.A. (2016). Automated integration of continuous glucose monitor data in the electronic health record using consumer technology. J. Am. Med. Inform. Assoc..

[7] Pagkalos I., Petrou L. (2016). SENHANCE: A Semantic Web framework for integrating social and hardware sensors in e-Health. Health Inform. J..

[8] Vuorimaa P., Harmo P., Hämäläinen M., Itälä T., Miettinen R. (2012). Active Life Home: A Portal-Based Home Care Platform. Proceedings of the 5th International Conference on PErvasive Technologies Related to Assistive Environments (PETRA ’12).

[9] Gay V., Leijdekkers P. (2015). Bringing Health and Fitness Data Together for Connected Health Care: Mobile Apps as Enablers of Interoperability. J. Med. Intern. Res..

[10] Wu W., Pirbhulal S., Zhang H., Mukhopadhyay S.C. (2019). Quantitative Assessment for Self-Tracking of Acute Stress based on Triangulation Principle in a Wearable Sensor System. IEEE J. Biomed. Health Inform..

[11] Hristoskova A., Sakkalis V., Zacharioudakis G., Tsiknakis M., De Turck F., Hristoskova A., Sakkalis V., Zacharioudakis G., Tsiknakis M., De Turck F. (2014). Ontology-Driven Monitoring of Patient’s Vital Signs Enabling Personalized Medical Detection and Alert. Sensors.

[12] Rea S., Pathak J., Savova G., Oniki T.A., Westberg L., Beebe C.E., Tao C., Parker C.G., Haug P.J., Huff S.M. (2012). Building a robust, scalable and standards-driven infrastructure for secondary use of EHR data: The SHARPn project. J. Biomed. Inform..

[13] Philip N., Butt T., Sobnath D., Kayyali R., Nabhani-Gebara S., Pierscionek B., Chouvarda I., Kilintis V., Natsiavas P., Maglaveras N. Design of a RESTful middleware to enable a web of medical things. Proceedings of the 2014 4th International Conference on Wireless Mobile Communication and Healthcare—”Transforming Healthcare Through Innovations in Mobile and Wireless Technologies” (MOBIHEALTH 2014).

[14] Bentley F., Tollmar K., Stephenson P., Levy L., Jones B., Robertson S., Price E., Catrambone R., Wilson J. (2013). Health Mashups: Presenting Statistical Patterns between Wellbeing Data and Context in Natural Language to Promote Behavior Change. ACM Trans. Comput.-Hum. Interact..

[15] Szilagyi I., Wira P. Ontologies and Semantic Web for the Internet of Things—A survey. Proceedings of the 42nd Annual Conference of the IEEE Industrial Electronics Society (IECON 2016).

[16] Liao C.F., Chen P.Y., Liao C.F., Chen P.Y. (2017). ROSA: Resource-Oriented Service Management Schemes for Web of Things in a Smart Home. Sensors.

[17] Guinard D., Trifa V. Towards the Web of Things: Web Mashups for Embedded Devices. Proceedings of the WWW 2009.

[18] Martins J.A., Mazayev A., Correia N. (2017). Hypermedia APIs for the Web of Things. IEEE Access.

[19] Wu Z., Xu Y., Yang Y., Zhang C., Zhu X., Ji Y., Wu Z., Xu Y., Yang Y., Zhang C. (2017). Towards a Semantic Web of Things: A Hybrid Semantic Annotation, Extraction, and Reasoning Framework for Cyber-Physical System. Sensors.

[20] HL7 Fast Healthcare Interoperability Resources (FHIR). https://hl7.org/fhir/.

[21] Bloomfield R.A., Polo-Wood F., Mandel J.C., Mandl K.D. (2017). Opening the Duke electronic health record to apps: Implementing SMART on FHIR. Int. J. Med. Inform..

[22] Luz M.P., Nogueira J.R.d.M., Cavalini L.T., Cook T.W. Providing Full Semantic Interoperability for the Fast Healthcare Interoperability Resources Schemas with Resource Description Framework. Proceedings of the IEEE 2015 International Conference on Healthcare Informatics.

[23] Jara A.J., Olivieri A.C., Bocchi Y., Jung M., Kastner W., Skarmeta A.F. (2014). Semantic Web of Things: An analysis of the application semantics for the IoT moving towards the IoT convergence. Int. J. Web Grid Serv..

[24] Patel P., Gyrard A., Datta S.K., Ali M.I. SWoTSuite: A Toolkit for Prototyping End-to-End Semantic Web of Things Applications. Proceedings of the 26th International Conference on World Wide Web Companion.

[25] Schema.org Hosted Extension: iot. https://iot.schema.org/.

[26] Compton M., Barnaghi P., Bermudez L., García-Castro R., Corcho O., Cox S., Graybeal J., Hauswirth M., Henson C., Herzog A. (2012). The SSN ontology of the W3C semantic sensor network incubator group. Web Semant..

[27] W3C Recommendation Semantic Sensor Network Ontology. https://www.w3.org/TR/vocab-ssn/.

[28] W3C Working Draft Web of Things (WoT) Thing Description. https://www.w3.org/TR/wot-thing-description/.

[29] Peng C., Goswami P., Bai G. An Ontological Approach to Integrate Health Resources from Different Categories of Services. Proceedings of the Third International Conference on Informatics and Assistive Technologies for Health-Care, Medical Support and Wellbeing (IARIA).

[30] Eysenbach G. (2001). What is e-health?. J. Med. Intern. Res..

[31] Choi J., Ha M., Im J., Byun J., Kwon K., Yoon W., Kim D., Heo S., Kim D. The patient-centric mobile healthcare system enhancing sensor connectivity and data interoperability. Proceedings of the 2015 International Conference on Recent Advances in Internet of Things (RIoT 2015).

[32] Seo D., Yoo B., Ko H. (2018). Information fusion of heterogeneous sensors for enriched personal healthcare activity logging. Int. J. Ad Hoc Ubiquitous Comput..

[33] Brickley D., Miller L. (2010). FOAF Vocabulary Specification. http://xmlns.com/foaf/spec/.

[34] W3C Recommendation PROV-O: The PROV Ontology. https://www.w3.org/TR/prov-o/.

[35] Tilahun B., Kauppinen T., Keßler C., Fritz F. (2014). Design and development of a linked open data-based health information representation and visualization system: Potentials and preliminary evaluation. JMIR Med. Inform..

[36] Trinugroho Y.B.D., Reichert F., Fensli R. An Ontology-Enhanced SOA-Based Home Integration Platform for the Well-Being of Inhabitants. Proceedings of the 4th IADIS International Conference on e-Health.

[37] Reda R., Piccinini F., Carbonaro A. (2018). Towards Consistent Data Representation in the IoT Healthcare Landscape. Proceedings of the 2018 International Conference on Digital Health (DH ’18).

[38] Dimou A., Vander Sande M., Colpaert P., Verborgh R., Mannens E., de Walle R. RML: A generic language for integrated RDF mappings of heterogeneous data. Proceedings of the 7th Workshop on Linked Data on the Web.

[39] Rhayem A., Mhiri M.B.A., Gargouri F. HealthIoT ontology for data semantic representation and interpretation obtained from medical connected objects. Proceedings of the IEEE/ACS International Conference on Computer Systems and Applications (AICCSA).

[40] Fielding R.T. (2000). Architectural Styles and the Design of Network-Based Software Architectures. Ph.D. Thesis.

[41] Kim J., Choi S.C., Ahn I.Y., Sung N.M., Yun J., Kim J., Choi S.C., Ahn I.Y., Sung N.M., Yun J. (2016). From WSN towards WoT: Open API Scheme Based on oneM2M Platforms. Sensors.

[42] Berners-Lee T. Linked Data—Design Issues. https://www.w3.org/DesignIssues/LinkedData.html.

[43] Alarcon R., Wilde E. Linking Data from RESTful Services. Proceedings of the Third Workshop on Linked Data on the Web.

[44] Lanthaler M., Gütl C. A semantic description language for RESTful Data Services to combat Semaphobia. Proceedings of the 5th IEEE International Conference on Digital Ecosystems and Technologies (IEEE DEST 2011).

[45] RDF Schema 1.1 | W3C. https://www.w3.org/TR/rdf-schema.

[46] OWL 2 Web Ontology Language | W3C. https://www.w3.org/TR/owl-syntax/.

[47] Peng C., Goswami P., Bai G. (2018). Linking Health Web Services as Resource Graph by Semantic REST Resource Tagging. Procedia Comput. Sci..

[48] Peng C., Bai G. Using Tag based Semantic Annotation to Empower Client and REST Service Interaction. Proceedings of the 3rd International Conference on Complexity, Future Information Systems and Risk.

[49] Lanthaler M., Gütl C. On using JSON-LD to create evolvable RESTful services. Proceedings of the Third International Workshop on RESTful Design (WS-REST ’12).

[50] Lucky M.N., Cremaschi M., Lodigiani B., Menolascina A., De Paoli F. (2016). Enriching API Descriptions by Adding API Profiles Through Semantic Annotation. Proceedings of the International Conference on Service-Oriented Computing.

[51] Panziera L., De Paoli F. A framework for self-descriptive RESTful services. Proceedings of the 22nd International Conference on World Wide Web (WWW ’13 Companion).

[52] Verborgh R., Steiner T., Van Deursen D., Van De Walle R., Valles J.G. Efficient runtime service discovery and consumption with hyperlinked RESTdesc. Proceedings of the 2011 7th International Conference on Next Generation Web Services Practices (NWeSP 2011).

[53] RDFLib/rdflib: A Python Library for Working With RDF, a Simple Yet Powerful Language for Representing Information. https://github.com/RDFLib/rdflib.

[54] Apache Jena—A Free and Open Source Java Framework for Building Semantic Web and Linked Data Applications. https://jena.apache.org/.

[55] HL7 FHIR Resource Observation—Examples. https://www.hl7.org/fhir/observation-examples.html.

